# Mid‐ to Long‐Term Follow‐Up Outcomes of Single Design Rotating Hinge Knee in Infected and Noninfected Revision Patients

**DOI:** 10.1111/os.70233

**Published:** 2026-01-11

**Authors:** Zhisen Gao, Tiejian Li, Ti Zhang, Minzhi Yang, Yonggang Zhou, Wei Chai

**Affiliations:** ^1^ Chinese PLA Medical School Beijing China; ^2^ Senior Department of Orthopedics The Fourth Medical Center of Chinese PLA General Hospital Beijing China; ^3^ National Clinical Research Center for Orthopedics, Sports Medicine and Rehabilitation Beijing China; ^4^ Department of Orthopedics Peking University Third Hospital Beijing China

**Keywords:** long‐term outcomes, prosthetic joint infection, revision total knee arthroplasty, rotating hinge knee

## Abstract

**Background:**

Total knee arthroplasty (TKA) offers significant relief for advanced knee osteoarthritis. With an aging population, TKA procedures are increasing, leading to a higher demand for revision surgeries. Rotating‐hinge knee (RHK) prostheses have emerged as a solution for complex revisions, but the long‐term durability of RHK prostheses and their effectiveness in infection‐related revisions remain controversial. Therefore, this study aimed to evaluate the mid‐ to long‐term clinical and survivorship outcomes of a single‐design rotating hinge knee (SDRHK) system in revision TKA, comparing patients revised for infection with those revised for noninfectious causes.

**Methods:**

This retrospective study analyzed 110 patients who underwent revision total knee arthroplasty (rTKA) with a SDRHK system from 2004 to 2023, with an average follow‐up of 11.3 years. Patients were divided into an infection group (*n* = 51) and a noninfection group (*n* = 59) for comparative analysis. Preoperative diagnostic arthrocentesis was performed to evaluate synovial cell count, leukocyte differential, and microorganisms. Functional outcomes were assessed using Hospital for Special Surgery (HSS) knee score, range of motion (ROM), and Knee Society Score (KSS). Study outcomes included prosthesis survival, mechanical failure, and complications. Data were analyzed using Kaplan–Meier survival analysis, *t* test, and *χ* ^2^ test, with statistical significance set at *p* ≤ 0.05.

**Result:**

The infection group experienced symptom onset significantly earlier than the noninfection group (18.8 vs. 50 months, *p* = 0.003), had a shorter initial prosthesis lifespan (32.7 vs. 66.8 months, *p* = 0.001), and underwent more surgeries before revision (2.6 vs. 1.6, *p* = 0.004). Microbiological analysis indicated that coagulase‐negative staphylococci and *Staphylococcus aureus* were the most commonly isolated pathogens. The 5‐ and 10‐year prosthesis survival rates in the infection group were 78.4% and 71%, respectively, while those in the noninfection group were 83.1% and 74.6%. At the latest follow‐up, survival rates for the two groups were 68.6% and 71.2%, showing similar outcomes. Functional scores in both groups improved postoperatively, with no significant differences in HSS, ROM, or KSS scores between the groups.

**Conclusion:**

This study highlights the important value of RHK prostheses in the treatment of prosthetic joint infection (PJI) after TKA. Despite challenges such as earlier symptom onset, shorter prosthesis lifespan, and higher complication rates in the infection group, their functional outcomes and prosthesis survival rates were comparable to those of the noninfection group, further validating the effectiveness of RHK prostheses. These findings provide useful references for clinical management of PJI and underscore the importance of continued innovation in revision techniques.

## Graphical abstract

RHK prostheses are effective for revision TKA in cases of infection, instability, or loosening after primary TKA, with mid‐ to long‐term follow‐up showing good functional recovery and high implant survival.

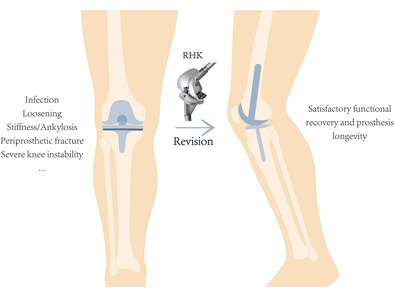

## 1 Introduction

Total knee arthroplasty (TKA) serves as a cornerstone in the management of advanced knee osteoarthritis, providing substantial pain relief and marked functional improvement for patients [1, 2]. As the global demographic shifts towards an aging population and the incidence of osteoarthritis escalates, TKA procedures have become increasingly prevalent, witnessing a predicted rise in revision TKA (rTKA) burden, with re‐revision rates reported as high as 18.98% at 15 years following the first rTKA in some regions [3, 4, 5]. Moreover, rTKA often presents considerable challenges, including severe bone loss, ligamentous instability, and compromised periprosthetic conditions [6, 7, 8]. The complexity of rTKA and the high complication rates, ranging from 9.2% to 63% [9], underscore the need for robust and versatile solutions.

Initially developed for tumor reconstruction, the rotating hinge knee (RHK) prosthesis has evolved into a crucial option in revision TKA, offering reliable stability in cases of severe bone loss and joint instability where conventional prostheses are insufficient [10, 11]. With continuous improvements in RHK prosthetic design, its effectiveness in managing complex situations, such as extensive bone defects, has also improved [12, 13, 14]. However, the long‐term durability of RHK prostheses remains controversial, primarily due to the high complication rates, including aseptic loosening, periprosthetic fractures, and deep infections [15, 16]. Studies have shown that the five‐year survival rate of RHKs varies significantly, ranging from 52% to 90% [17, 18, 19].

In particular, patients with a history of infection often experience higher complication rates, and their prosthesis survival rates are typically lower than those of noninfected patients. Previous studies have indicated that the infection status at the time of revision surgery can significantly impact prognosis, thereby affecting the longevity of the prosthesis and the functional recovery of the patient [20, 21, 22]. However, there is limited research specifically comparing the outcomes of single‐design RHK prostheses in infected versus noninfected patients in revision TKA.

This study aims to evaluate the mid to long‐term outcomes of a single‐design RHK prosthesis in revision TKA patients, with a focus on comparing outcomes between infected and noninfected cases. By analyzing survival rates, functional outcomes, and complication profiles, this research seeks to provide a clearer understanding of the efficacy of RHKs in complex revision scenarios.

## 2 Methods

### 2.1 Study Design and Ethical Considerations

This single‐center retrospective study was approved by the Hospital (2025KY016‐KS001). The informed consent was obtained from the patients in the study. The study spanned from January 2004 to December 2023, focusing on patients who underwent rTKA using the single design rotating hinge knee (SDRHK) system. The research plan was crafted to strictly follow the highest ethical guidelines in medical research.

### 2.2 Patient Cohort and Data Collection

A total of 160 patients who received RHK prosthesis implantation were retrospectively evaluated. Exclusion criteria included patients who required endoprostheses with distal femoral replacements or those with bone tumors. Additionally, patients who underwent primary complex knee replacement using an RHK were excluded. Unfortunately, 13 participants passed away due to nonsurgical, noninfectious causes, and 37 participants were lost to follow‐up after losing contact. Ultimately, 110 patients were available for the final analysis.

To aid in the diagnosis, preoperative diagnostic arthrocentesis was performed in patients to analyze the cell count, differential leukocyte count, and microorganisms in the synovial fluid [23]. Additionally, white blood cell count and C‐reactive protein levels were routinely measured according to the IDSA guidelines [24]. The study patients had a mean follow‐up time of 11.3 years (range, 5.5–21 years). Among them, 59 patients underwent revision surgeries primarily due to instability or aseptic loosening, while 51 patients required revisions due to periprosthetic joint infections (PJI).

### 2.3 Surgical Procedure

**FIGURE 1:**
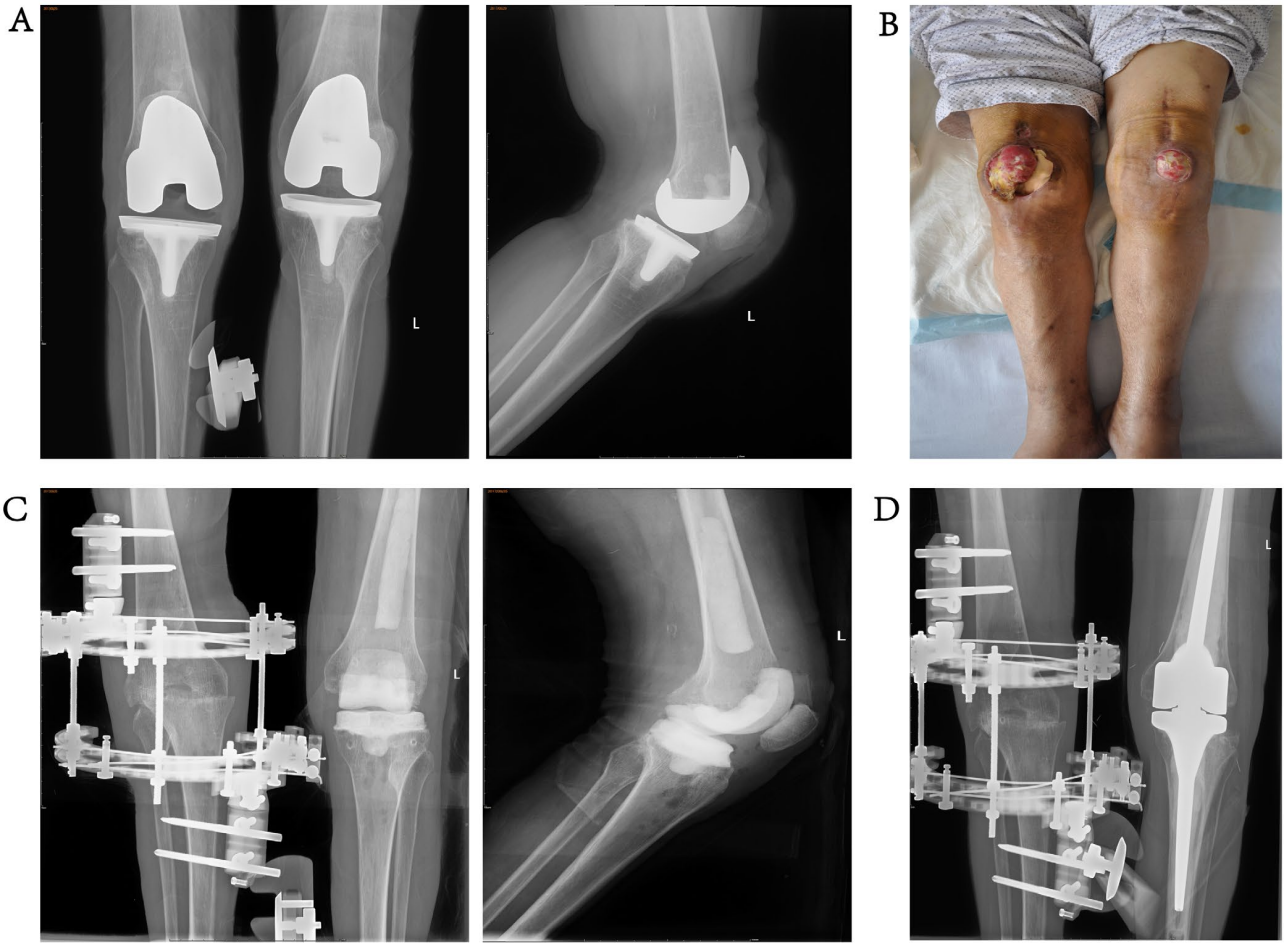
Case images of treatment following infection after TKA: (A) radiograph after primary TKA; (B) clinical appearance of the knee after infection; (C) postoperative radiograph after the first surgery for infection treatment; and (D) radiograph after implantation of a left RHK prosthesis.

Each operation utilized the intricately designed SDRHK system. The prosthesis used in the procedure is the endo‐model rotating‐hinge prosthesis (Waldemar Link GmbH & Co. KG, Hamburg, Germany). This prosthesis is a fully cemented, nonmodular design with long intramedullary stems, a patellar flange, anti‐luxation device, and rotational feature, chosen for its outstanding durability and motion flexibility. For revision surgery in cases diagnosed with PJI, the detailed surgical procedure followed the protocol as described in the previous publication [25]. The standard medial parapatellar arthrotomy technique provided optimal joint access while effectively preserving the integrity of the surrounding tissues. During the procedure, existing components were removed, followed by comprehensive debridement, a crucial step for ensuring successful prosthesisation (Figure 1). The reconstruction phase emphasized restoring the knee joint line for biomechanical alignment and function, achieved through anatomical markers and soft‐tissue tension measurements. The SDRHK system's features, including its rotational hinge mechanism, facilitated controlled knee movement postoperatively. The system's modularity and advanced surgical instruments enabled precise Prosthesis positioning, enhancing fit and minimizing postoperative complications such as aseptic loosening.

### 2.4 Follow‐Up and Data Collection

Postoperative follow‐up was conducted at 3 months, 12 months, and then annually. The patient follow‐up methods included outpatient visits, telephone follow‐ups, or communication via WeChat. Radiographic evaluations were conducted either at the local hospital or at our outpatient clinic. Additionally, the patient's gait, range of motion (ROM), and activities such as squatting, stair climbing, and descending were documented through video recordings. Data collection included baseline demographic statistics, medical history, perioperative details, and contact information. To assess functional outcomes, we relied on patient‐reported outcome measures (PROMs). PROMs like the Hospital for Special Surgery (HSS) knee score, ROM, and the Knee Society Score (KSS) were employed for functional assessment.

### 2.5 Outcome Measures

The primary outcome measures of this study were focused on evaluating prosthesis survivorship and mechanical failure, defined by the presence of progressive radiolucent lines or necessitating revision surgery. Secondary outcome measures included a range of complications, namely recurrent infection, prosthesis dislocation, patellar dislocation, extension lag, and unexplained pain. Furthermore, data on all forms of reoperations and revisions, irrespective of their underlying cause, were meticulously recorded.

### 2.6 Clinical Case and Radiographic Assessments

Preoperative and postoperative radiographs were evaluated using standardized protocols including long‐leg views, and assessment of bone loss (Figure 1). Clinical evaluations involved evaluation of self‐sufficiency and knee function using standardized scoring systems. The compilation of these outcomes was carried out through a comprehensive and multifaceted approach. For patients who no longer visited the clinic, we gathered information through phone calls and wechat. Our team meticulously reviewed electronic patient records in great detail to guarantee both accuracy and completeness of the data. Additionally, we performed an in‐depth analysis of information retrieved from electronic medical records (EMR) system, aiming to offer a broader and more comprehensive context for our findings.

### 2.7 Statistical Analysis

Data were analyzed using R v4.1.0 (R Foundation for Statistical Computing, Vienna, Austria), SPSS v26 (IBM Corp., Armonk, NY, USA), and GraphPad Prism 9 (GraphPad Software, San Diego, CA, USA). Kaplan–Meier survival analysis was performed for prosthesis survivorship. We utilized descriptive statistics to analyze the data distribution. For group comparisons, we applied *t* tests and *χ* ^2^ tests. Statistical significance was set at *p* ≤ 0.05.

## 3 Results

### 3.1 Patient Demographics and Indications

In this investigation, the research encompassed two groups: 51 patients undergoing knee revision surgery due to infectious causes and 59 patients due to noninfectious causes (Table 1). A comparative analysis between these cohorts revealed no statistically significant differences in several key demographic and health‐related parameters. Specifically, there were no significant differences in gender distribution (*p* = 0.122), age profile (*p* = 0.059), body mass index (BMI) (*p* = 0.120), the frequency of preoperative complications, or the American Society of Anesthesiologists (ASA) scores (*p* = 0.235). In the McPherson grading system, there was a statistically significant difference in the local extremity grade between the infection group and the noninfection group (*p* = 0.005). This indicates that patients in the infection group were clearly in a state of PJI.

**TABLE 1:** Patient baseline demographics.

Characteristics	Infection (*n* = 51)	Noninfection (*n* = 59)	*p*
Gender			
Male	16 (31.4%)	11 (18.6%)	0.122
Female	35 (68.6%)	48 (81.4%)	
Age (years)	66.3 ± 10.9	61.7 ± 14.6	0.059
BMI (kg/m^2^)	27.2 ± 4	25.9 ± 5	0.120
Chronic disease			
Hypertension	22 (43.1%)	26 (51%)	0.922
Cardiopathy	10 (19.6%)	7 (11.9%)	0.263
Diabetes mellitus	7 (13.7%)	11 (18.6%)	0.487
Rheumatoid arthritis	2 (3.9%)	8 (13.6%)	0.080
Other	13 (25.5%)	19 (32.2%)	0.439
ASA grade			
I	4 (7.8%)	11 (18.6%)	0.235
II	36 (70.6%)	37 (62.7%)	
III	11 (21.6%)	10 (17%)	
IV	0	1 (1.7%)	
Mcpherson grade (systemic host grade)			
A	30 (58.8%)	30 (50.9%)	0.671
B	20 (39.2%)	27 (45.8%)	
C	1 (2%)	2 (3.4%)	
Mcpherson grade (local extremity grade)			
1	10 (19.6%)	29 (49.2%)	0.005
2	34 (66.7%)	26 (44.1%)	
3	7 (13.7%)	4 (6.8%)	
Smoking	5 (9.8%)	3 (5.1%)	0.560
Drinking	2 (3.9%)	2 (3.4%)	0.882

Abbreviations: ASA, American Society of Anesthesiology; BMI, body mass index.

A breakdown of the specific reasons necessitating rTKA in the entire cohort of 110 patients revealed a range of clinical indications. This included 51 infection‐driven revisions (46.4%); the remaining 59 patients (53.6%) were noninfected, with reasons for revision including 16 cases (14.5%) of aseptic loosening of the prosthesis, 7 cases (6.4%) of severe knee instability, 11 cases (10%) of stiffness or ankylosis, 10 cases (9.1%) of periprosthetic fractures, 5 cases (4.5%) of unexplained pain, 4 cases (3.6%) of prosthesis fracture, 2 cases (1.8%) of liner wear, 2 cases (1.8%) due to hyperextension complications, and 2 cases (1.8%) due to prosthesis dislocation (Table 2).

**TABLE 2:** Indications for revision of rotary hinge knee arthroplasty.

Indications for rTKA	*N* (%)
Infection	51 (46.4%)
Loosening (femoral/tibial)	16 (14.5%)
Stiffness/ankylosis	11 (10.0%)
Periprosthetic fracture	10 (9.1%)
Severe knee instability	7 (6.4%)
Unexplained pain	5 (4.5%)
Prosthesis fracture	4 (3.6%)
Hyperextension	2 (1.8%)
Liner wear	2 (1.8%)
Prosthesis dislocation	2 (1.8%)

Abbreviation: rTKA, revision total knee arthroplasty.

### 3.2 Details of Rotary Hinge Knee Prosthesis Revision Surgery

We conducted a detailed comparison between patients undergoing knee revision surgery due to infection and those due to noninfectious causes (Table 3). No statistically significant difference was observed in the reasons for the initial TKA between the infection group and those in the noninfection group (*p* = 0.277). However, the infection group experienced symptoms markedly sooner (18.8 ± 11.9 months) compared to their noninfection counterparts (49.9 ± 15.8 months, *p* = 0.003), indicating a propensity for earlier postoperative infection manifestation. This earlier onset of symptoms in the infection group consequently correlated with a markedly shorter lifespan of their initial TKA prostheses, standing at 32.7 ± 13.1 months. This was significantly lower than the noninfection group, which had a prosthesis lifespan of 66.8 ± 15.7 months (*p* = 0.001).

**TABLE 3:** Details of rotary hinge knee prosthesis revision surgery.

	Infection (*n* = 51)	Noninfection (*n* = 59)	*p*
Reasons for primary TKA (excluding tumors)			
Osteoarthritis (OA)	43 (84.3%)	42 (71.2%)	
Rheumatoid arthritis (RA)	3 (5.9%)	8 (13.6%)	
Ankylosing spondylitis	1 (2%)	4 (6.8%)	
Other	4 (7.8%)	5 (8.5%)	
Months to symptom onset (from primary TKA) (months; SD)	18.8 ± 11.9	49.9 ± 15.8	0.003**
Duration of symptoms (weeks; SD)	80.2 ± 31.28	64.1 ± 23.6	0.405
Months from onset of symptoms to revision (months; SD)	13.9 ± 4.4	16.8 ± 6.4	0.465
First prosthesis life (months; SD)	32.7 ± 13.1	66.8 ± 15.7	0.001**
Number of operations before revision (SD)	2.6 ± 0.3	1.6 ± 0.3	0.004
Collateral ligament function			
Normal	21 (23.5%)	26 (44.1%)	0.947
Injured	27 (52.9%)	30 (50.9%)	
Ruptured	3 (5.9%)	3 (5.1%)	
Bleeding (mL; SD)	755.9 ± 118.1	695.1 ± 137.3	0.161
Transfusion			
Red blood cell (U; SD)	3.5 ± 0.6	3.9 ± 0.8	0.380
Plasma (U; SD)	2.5 ± 0.6	2.5 ± 0.5	0.723
Anderson Orthopaedic Research Institute classification (femur)			
0	12 (23.5%)	26 (44.1%)	0.079
1	15 (29.4%)	12 (20.3%)	
2	23 (45.1%)	21 (35.6%)	
3	1 (2%)	0	
Anderson Orthopaedic Research Institute classification (tibia)			
0	12 (23.5%)	28 (47.5%)	0.032*
1	26 (51%)	19 (32.2%)	
2	12 (23.5%)	12 (20.3%)	
3	1 (2%)	0	
Prosthesis size			
Large	5 (9.8%)	2 (3.4%)	0.381
Medium	27 (52.9%)	34 (57.6%)	
Small	19 (37.2%)	23 (39%)	

*Note*: *p < 0.05, **p < 0.01.

The number of operations before revision was notably higher in the infection group than in the noninfection group (2.6 ± 0.3 vs. 1.6 ± 0.3, *p* = 0.004). This could be attributed to the necessity for multiple revisions, debridement, or replacement of insert typically required in the infected group. In terms of bone defects, the infection group demonstrated a more pronounced degree of tibial bone loss compared to their noninfection counterparts (*p* = 0.032). The type of prosthesis used did not show a significant impact on the incidence of infection (*p* = 0.381).

Bacteria were isolated in 38 patients (74.5%), with 16 testing positive for coagulase‐negative Staphylococci and 7 for *S. aureus* , along with other bacterial species. In cases where no bacteria were detected (false negative results), additional parameters based on the International Consensus Meeting criteria were used to confirm the diagnosis of PJI. Coagulase‐negative staphylococci were recognized as the predominant pathogen detected, observed in the synovial fluid of 16 individuals (31.4%). This was followed by *S. aureus* (13.7%), *Enterococcus faecalis* (7.8%), fungi (5.9%), *Klebsiella oxytoca* (3.9%), Streptococcus (2%), and other pathogens (9.8%). Furthermore, six patients exhibited a sinus tract. Regarding therapeutic strategies, the most prevalent approach entailed conducting two revision surgeries, selected for 24 patients (47.1%) within the infection cohort (Table 4).

**TABLE 4:** Information of patients with infection‐related revisions.

Variable	*N* (%)
Bacterial species	
Coagulase‐negative Staphylococci	16 (31.4%)
Staphylococci	
*Staphylococcus aureus*	7 (13.7%)
*Enterococcus faecalis*	4 (7.8%)
Fungi	3 (5.9%)
*Klebsiella oxytoca*	2 (3.9%)
Streptococcus	1 (2%)
Other	5 (9.8%)
Inflammatory markers (SD)	
ESR (mm/h)	27.7 ± 10.6
CRP (mg/dL)	1.8 ± 1.1
IL‐6 (pg/mL)	19.6 ± 14.3
d‐dimer (μg/mL)	4 ± 2.4
Fibri (g/L)	4.4 ± 0.8
Sinus tract	6 (11.8%)
Treatment method	
DAIR	10 (19.6%)
One‐stage revision	17 (33.3%)
Two‐stage revision	24 (47.1%)
Amputation	0

Abbreviation: DAIR, debridement, antimicrobial therapy, and implant retention.

### 3.3 Outcomes of Infection Versus Noninfection Cohorts After SDRHK Surgery

**FIGURE 2:**
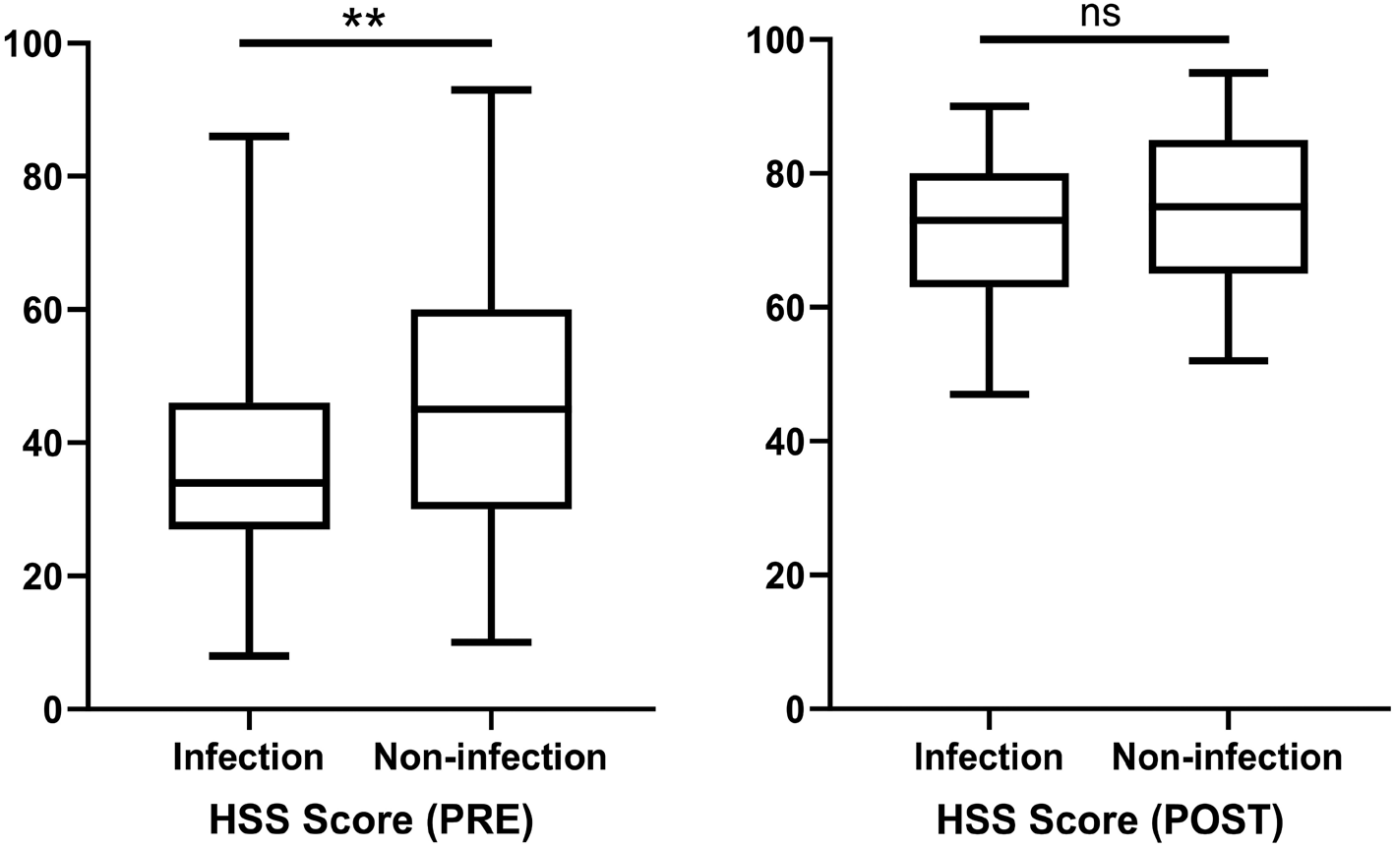
Preoperative and postoperative scores of HSS (**p < 0.01; ns means no statistical significance).

**FIGURE 3:**
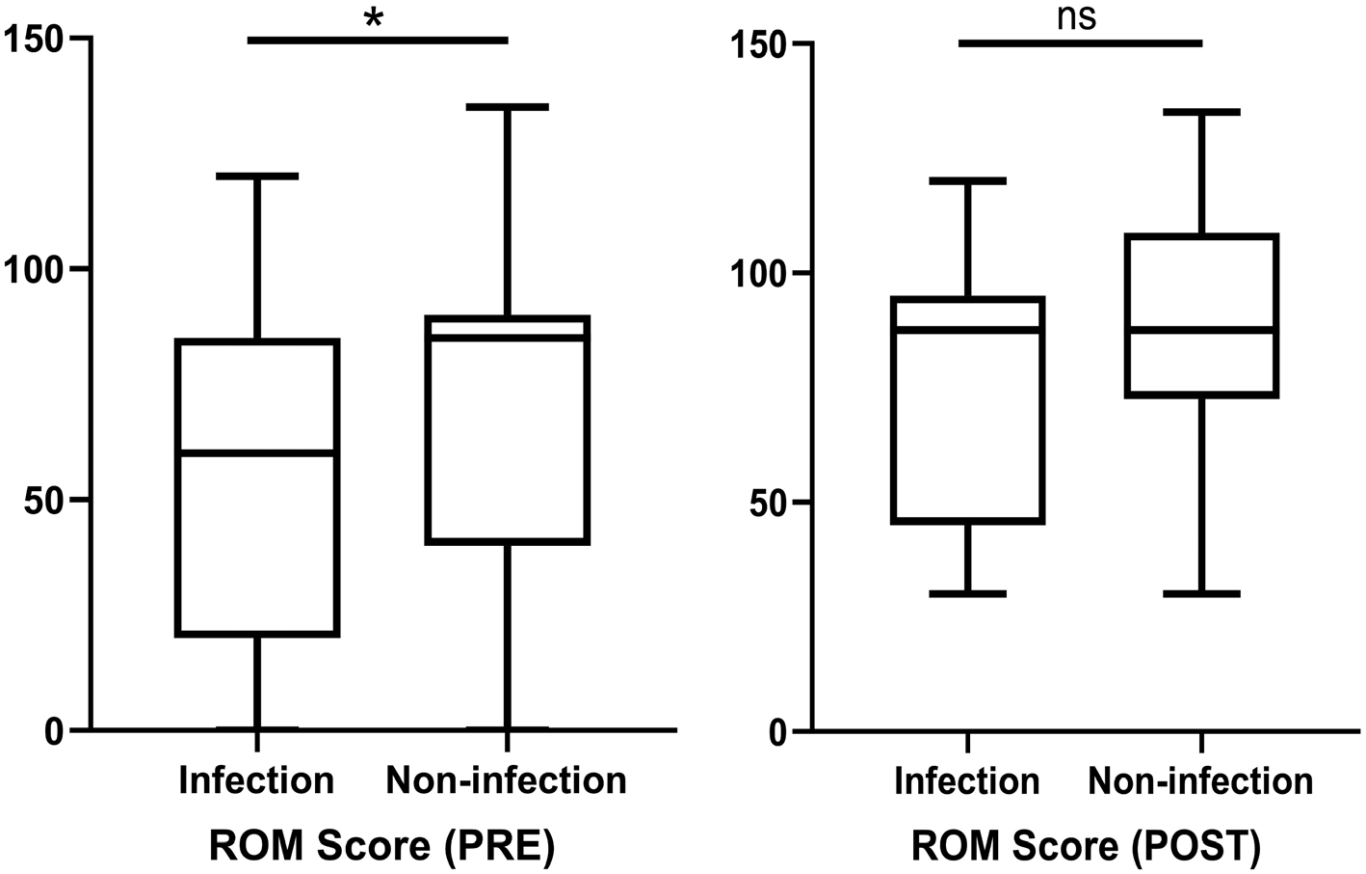
Preoperative and postoperative scores of ROM (*p < 0.05; ns means no statistical significance).

**FIGURE 4:**
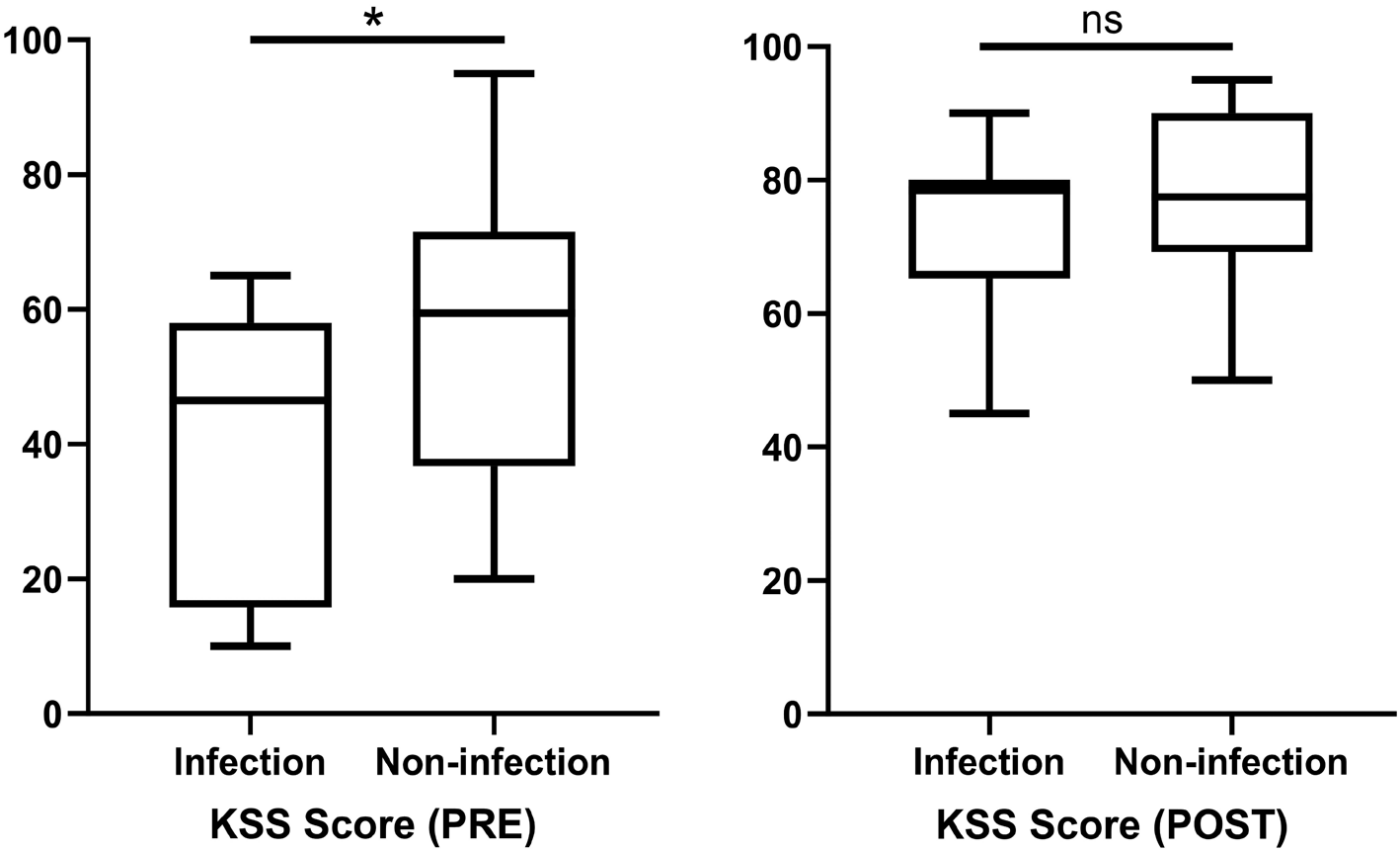
Preoperative and postoperative scores of KSS (*p < 0.05; ns means no statistical significance).

During the SDRHK procedure, patients in the infection cohort had significantly longer hospitalization durations compared to the noninfection group (2.6 ± 0.3 vs. 1.6 ± 0.3, *p* = 0.004). Preoperatively, the infection group demonstrated significantly lower HSS, ROM, and KSS scores compared to the noninfection group (Table 5). Postoperatively, no significant differences were observed between the infection and noninfection groups in terms of HSS, ROM, or KSS scores (Table 5). Figures 2, 3, 4 provide a more visual representation of these results, illustrating the preoperative and postoperative scores for both groups through box plots, which highlight the improvements in both cohorts, though no significant differences were observed after surgery.

**TABLE 5:** Preoperative and postoperative rehabilitation and follow‐up information.

Variable	Infection (*n* = 51)	Noninfection (*n* = 59)	*p*
Hospitalization days (SD)	23.6 ± 3.1	19.5 ± 3.9	0.003**
HSS score (SD)			
Preoperative	37.8 ± 4.9	48.2 ± 5.6	0.010*
Postoperative	73.9 ± 2.9	75.1 ± 6.8	0.434
ROM score (°; SD)			
Preoperative	57 ± 10.4	70.9 ± 9.4	0.036*
Postoperative	76.9 ± 14.2	89.1 ± 14	0.315
KSS score			
Preoperative	40.3 ± 10.1	56.4 ± 11.5	0.042*
Postoperative	73.5 ± 6	77.8 ± 6.5	0.290
Follow‐up duration (years; SD)	8.9 ± 1.2	9.2 ± 1.1	0.661
Satisfaction	40 (78.4%)	49 (83.1%)	0.539
Survival rate of 5 years	78.4%	83.1%	
Survival rate of 10 years	71%	74.6%	
Complications			
Recurrent infection	5 (9.8%)	3 (5.1%)	0.560
Prosthesis dislocation	4 (7.8%)	2 (3.4%)	0.303
Patellar dislocation	0	1 (1.7%)	0.263
Extension lag	2 (3.9%)	5 (8.5%)	0.559
Unexplained pain	6 (11.8%)	8 (13.6%)	0.778

*Note*: *p < 0.05, **p < 0.01.

Abbreviations: HSS, Hospital for Special Surgery knee score; KSS, Knee Society Score; ROM, range of motion.

**FIGURE 5:**
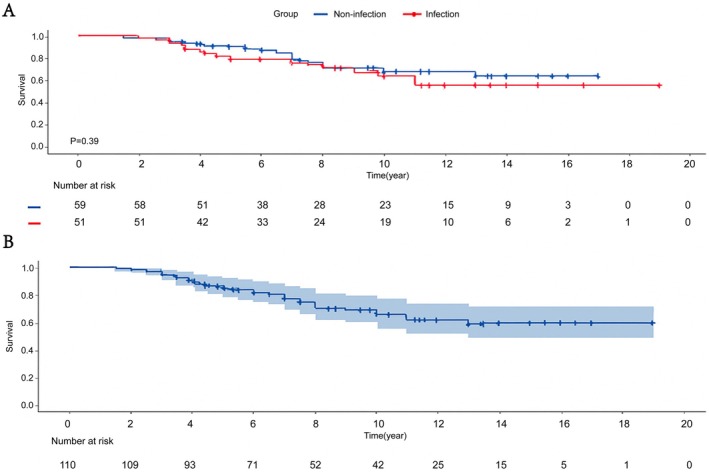
(A) Prosthetic survival curve and risk prediction in infection and noninfection groups. (B) Prosthetic survival curve and risk prediction for the whole patient population.

The mean follow‐up period of the infection group was 10.9 ± 1.2 years, while that of the noninfection group was 11.2 ± 1.1 years. The 5‐year prosthesis survival rate was 78.4% for the infection group and 83.1% for the noninfection group, while the 10‐year prosthesis survival rate was 71% for the infection group and 74.6% for the noninfection group (Table 5). Until the latest follow‐up date, the prosthesis survival rate was 68.6% for the infection group and 71.2% for the noninfection group, as illustrated by the prosthesis survival curves (Figure 5A). Figure 5B displays the survival curve for all patients, highlighting the overall trend in survival over time.

Both groups also experienced complications after the revision surgery. These complications included recurrent infections (5 cases in the infection group vs. 3 in the noninfection group), prosthesis dislocations (4 vs. 2), patellar dislocations (0 vs. 1), extension lag (2 vs. 5), and unexplained pain (6 vs. 8). Most recurrent cases were treated with DAIR (debridement, antibiotics, and implant retention) or long‐term suppressive antibiotic therapy. Other complications were managed symptomatically without additional surgical intervention. Overall, most patients achieved stable symptom control during follow‐up.

## 4 Discussion

### 4.1 Impact of PJI on the Clinical Course and Surgical Complexity of Revision TKA

This study provides a comprehensive comparison of knee revision surgery outcomes between patients with PJI and those with noninfectious causes, revealing critical insights into the unique challenges posed by PJI. Our findings demonstrate that patients with PJI not only exhibit an earlier onset of symptoms but also experience a significantly shorter lifespan of their initial prostheses. Additionally, the infection group had longer hospitalization durations compared to the noninfection group. These observations highlight the aggressive nature of infections and their profound impact on the longevity and management of knee prosthesis.

The significantly shorter time interval between the initial TKA and symptom onset in the infection group (18.8 ± 11.9 months) compared to the noninfection group (49.9 ± 15.8 months) underscores the early and often rapid manifestation of PJI. This early onset correlates with a notably reduced prosthesis lifespan in the infection group (32.7 ± 13.1 vs. 66.8 ± 15.7 months in the noninfection group), reflecting the destructive impact of infection on prosthetic joints. The literature consistently supports this observation, where infections are recognized as a leading cause of early prosthesis failure due to the inflammatory processes that compromise prosthesis stability [26, 27, 28].

Furthermore, the infection group required significantly more surgical interventions prior to the definitive revision, with an average of 2.6 ± 0.3 operations compared to 1.6 ± 0.3 in the noninfection group. This increase in surgical burden is indicative of the complexities in managing PJI, where multiple debridements, exchange of components, and management of persistent infection are often necessary. In rTKA, bone defects are commonly observed on the medial and lateral sides of the tibia [29]. Additionally, the more severe tibial bone defects observed in the infection group highlight the extent of bone loss associated with chronic infections, which further complicates revision procedures and may necessitate the use of more complex reconstructive techniques.

### 4.2 Microbiological Profile and Infection Burden in PJI Revisions

The most commonly isolated bacteria in PJI are typically *Staphylococcus epidermidis* and *S. aureus* . However, other pathogens, including methicillin‐resistant *Staphylococcus aureus* (MRSA) and Gram‐negative bacteria such as *Pseudomonas aeruginosa* , may also be found [30, 31, 32, 33]. The microbiological profile observed in our infection cohort, dominated by coagulase‐negative staphylococci and *S. aureus* , mirrors the patterns seen in other studies on PJI [34, 35, 36], emphasizing the persistent challenge these pathogens present. The presence of multiple pathogens and the occurrence of sinus tracts further complicate the management of these cases, often requiring a more aggressive surgical approach and longer hospitalization, as seen in our infection group. The high prevalence of these pathogens, coupled with the frequent need for two‐stage revision surgeries in our infection cohort, further emphasizes the importance of targeted antimicrobial therapy and a staged surgical approach for the effective management of PJI. Comparing these findings to other studies in the field underscores the ongoing challenge of managing PJI and highlights the need for tailored treatment strategies based on the microbiological profile of the infection.

### 4.3 Functional Outcomes of SDRHK in Infected vs. Noninfected Revisions

Chen et al. [27] reported the functional outcomes of 31 patients who underwent two‐stage septic revision TKA, with an average KSS score of 70.7. Schnetz et al.'s study showed that patients who received a hinged prosthesis for multistage revision knee arthroplasty due to severe periprosthetic joint infection had an average KSS of 74.3 (range, 24–99) [34]. Additionally, Cottino et al.'s research indicated that the average KSS score following complex primary and two‐stage revision with a rotating hinge prosthesis was around 81 [37]. These results are similar to our study, where functional improvement was observed after the use of the RHK prosthesis. However, these studies either focused solely on infected patients or combined data from both infected and noninfected patients. Our study not only confirms the effectiveness of the RHK prosthesis in managing complex revision surgeries but also further compares the functional differences between infected and noninfected patients. Our findings suggest that despite the higher complexity and complication rates associated with PJI, there were no significant differences in the improvement of functional outcomes, including ROM, HSS, and KSS, between infected and noninfected patients. This indicates that, even in high‐risk revision cases, the RHK prosthesis can still effectively restore knee function.

### 4.4 Mid‐ to Long‐Term Survivorship of SDRHK and Comparison With Previous Studies

Yoon et al. [38] compared the survival rates and outcomes of RHK and condylar constrained knee prostheses, and their analysis revealed an overall survival rate of 81.3% during mid‐term follow‐up (5–10 years). Eskelinen et al. [39] also reported a 10‐year prosthesis survival rate of 81.7% for RHK prostheses. While these survival rates are slightly higher than our 10‐year prosthesis survival rate, it is important to note that the number of infected patients included in these studies was relatively low. Another study, which included 131 patients with either aseptic or infected revisions of RHK prostheses, reported a 5‐year survival rate of 72%, including 73 cases of infected revisions [18], which is similar to our prosthesis survival rate.

In our study, the 5‐year and 10‐year prosthesis survival rates for the infected group were 78.4% and 71%, respectively, while the noninfected group had survival rates of 83.1% and 74.6%. At the most recent follow‐up, the Prosthesis survival rates for the infected and noninfected groups were 68.6% and 71.2%, respectively. The survival rates between the two groups were comparable, and there was no significant difference in complications. This may be attributed to the fact that RHK prostheses do not heavily rely on soft tissue and ligament support, as they provide adequate stability and functional foundation on their own. Additionally, thorough debridement during infection management likely contributed to the similar outcomes in terms of both Prosthesis survival and complications between the two groups.

### 4.5 Strengths and Limitations

This study has several strengths. It represents a relatively large, single‐center cohort of patients undergoing revision TKA with a SDRHK system and a mean follow‐up of 11.3 years, allowing robust assessment of mid‐ to long‐term outcomes. The inclusion of both infection‐ and noninfection–related revisions enables a direct comparison of clinical course, functional recovery, and prosthesis survivorship between these two clinically relevant subgroups. This study also has several limitations that should be acknowledged. Firstly, the retrospective design inherently introduces selection biases and limits the ability to establish causal relationships. Secondly, the relatively small sample size may affect the generalizability of our findings, particularly concerning the long‐term outcomes of RHK prostheses in infection‐related revisions. Additionally, while the follow‐up period was substantial, it may not fully capture all late‐stage complications, especially in cases of chronic PJI. Future studies should aim to include larger patient cohorts and longer follow‐up periods to provide a more comprehensive understanding of the long‐term performance of RHK prostheses in complex revision scenarios.

## 5 Conclusion

In conclusion, our study highlights the critical role of RHK prostheses in the treatment of PJI following TKA. The infection group showed earlier onset of symptoms, shorter prosthesis lifespan, and higher complication rates, emphasizing the complexity of managing PJI. Despite these challenges, the functional outcomes and prosthesis survival rates after revision were comparable between the infection and noninfection groups, further confirming the effectiveness of the RHK prosthesis. These findings provide valuable insights into the management of PJI and underscore the need for continuous innovation and optimization in revision joint arthroplasty techniques.

## Author Contributions

**Tiejian Li**, **Zhisen Gao**, and **Wei Chai:** conceptualization. **Tiejian Li**, **Zhisen Gao**, and **Ti Zhang:** methodology. **Tiejian Li** and **Minzhi Yang:** investigation. **Tiejian Li** and **Minzhi Yang:** formal analysis. **Tiejian Li** and **Zhisen Gao:** writing – original draft. **Tiejian Li**, **Zhisen Gao**, **Yonggang Zhou**, and **Wei Chai:** writing – review and editing. **Wei Chai:** funding acquisition, supervision, resources.

## Funding

This work was supported by the Beijing Science and Technology Plan “AI+ Health Collaborative Innovation Cultivation” project (No. Z221100003522014).

## Disclosure

All authors listed meet the authorship criteria according to the latest guidelines of the International Committee of Medical Journal Editors. All authors are in agreement with the manuscript.

## Conflicts of Interest

The authors declare no conflicts of interest.

## Data Availability

The data that support the findings of this study are available on request from the corresponding author. The data are not publicly available due to privacy or ethical restrictions.
